# Précis of *Inducing Immunity? Justifying Immunization Policies in Times of Vaccine Hesitancy*  ^1^

**DOI:** 10.1093/phe/phag016

**Published:** 2026-07-13

**Authors:** Roland Pierik, Marcel Verweij

**Affiliations:** Department of Foundations of Law, Maastricht University, The Netherlands; Ethics Institute, Department of Philosophy and Religious Studies, Utrecht University, The Netherlands

1. Vaccination programmes are very effective measures for protecting against outbreaks of certain serious infectious diseases like measles, poliomyelitis, and diphtheria. This is because immunisations not only protect persons against permanently disabling or even potentially fatal diseases but also prevent them from spreading infections to others.

2. Governments have two main responsibilities in relation to immunisation. One is to protect public health and societal life. The other is to secure equitable access to essential vaccines for all persons. Both responsibilities are reasons for governments to aim for a level of vaccine coverage in the population sufficient for group-level protection, sometimes called ‘herd immunity’. If group-level protection is secured, infectious agents cannot spread through the population. Initial outbreaks will soon fade, and even unvaccinated individuals will be well protected.

3. Although a solid majority of citizens in most countries routinely take part in immunisation programmes, significant groups have doubts about participation, postpone doing so or even completely reject vaccination. Important grounds for such vaccine hesitancy and refusal include, among others, religious objections, views of life that embrace ‘living in line with nature’, concerns about side-effects, and outright distrust of the state.

4. In a constitutional democracy, the government cannot simply dismiss such concerns and require all citizens to be vaccinated. This would infringe fundamental rights, including the freedom of religion, conscience, and belief, and the right to bodily integrity. For parents who object to childhood immunisation, it would also violate their right to family life. Although these rights are fundamental, they are never absolute, and under certain conditions it can be proportionate to impose limits on them.

5. Our overall argument for limiting these rights and freedoms in the case of vaccination is the harm principle, eloquently laid out in John Stuart Mill’s *On Liberty* (Mill, 1859/2003) and further developed by Joel Feinberg in *Harm to Others* (Feinberg, 1984): it can be justified to curtail individual liberty if this is necessary to prevent harm to others. The harm principle, however, cannot be applied straightforwardly to vaccination programmes. After all, how would my unwillingness to be immunised, or that of my child, harm other people if they—or their parents—can opt for such protection themselves?

6. The first response is that many children are too young to be vaccinated (e.g. against measles) and are at risk when exposed. Other individuals have lost their immunity due to disease, immunosuppressive therapy, or simply ageing. These people depend on group-level protection, which requires a high immunisation rate. The government has a key responsibility to protect the basic interests of children and of other persons who cannot seek protection for themselves.

7. The most promising normative argument, therefore, is to acknowledge that vaccination refusal can undermine the collective protection that societies seek to establish against serious infectious diseases. If people can opt out without negative consequences, other vaccine-hesitant people will follow their example. In this way, vaccine refusal harms the collective endeavour to realise group-level protection against larger outbreaks, which will be especially dangerous for those who are vulnerable to infection—notably small children, who will often be most at risk.

This suffices to see vaccine refusal (either for oneself or for one’s child) as harmful to others, and thus as a choice that can be constrained by appealing to the harm principle.

8. The harm principle thus provides a basic justification for curtailing people’s freedom to opt out of vaccination programmes. However, for a mandatory vaccination policy to be justified, the infringement of freedom should also be in line with the principle of proportionality. This is especially relevant given that not every individual needs to be vaccinated. A coverage of around 90 to 95 per cent is sufficient to protect all—and this creates room for allowing refusal.

9. Therefore, even though democratic governments have an obligation to safeguard the basic interests of children and to intervene in family life if parents put those interests at risk, we do not think it is justified to force all parents to vaccinate their child. If overall vaccine coverage is sufficient for group-level protection, there is no legal basis for compelling parents to vaccinate their child.

10. When a proportionate policy is being developed, many contextual factors are relevant, including the current level of voluntary participation, the severity of the relevant diseases, the nature of the vaccines included in the programme, the nature and magnitude of the ‘force’ applied, and the remaining options for opting out.

11. For childhood immunisation, we recommend that democratic states that still have a voluntary system should enact a law specifying (i) a minimum vaccination rate that the government must maintain for the national programme, and (ii) the ways in which parental freedom to opt out will be curtailed if participation falls below this minimum. A reasonable measure following from this could be to require all children attending day care centres to be vaccinated, and, if needed, also those of school age.

12. We argue that a policy that makes immunisation mandatory while offering parents the option to apply for religious or ‘philosophical’ exemptions—as is common in most US states—cannot be justified in a constitutional democracy. For such rule-and-exemption policies to be feasible and justifiable, they must simultaneously satisfy various requirements, which appears impossible. Such policies will either lead to too many exemptions or unduly violate the democratic ideal of state neutrality by privileging or discriminating against certain religious or non-religious doctrines.

13. Until the COVID-19 pandemic, discussion about mandatory immunisation mostly concerned parental refusal to vaccinate their children. Can immunisation policies also set limits on people’s freedom to forgo vaccination *for themselves—*specifically in the context of a pandemic? Interestingly, the competing rights and interests on either side are more significant than in the case of childhood immunisation.

14. On the one hand, this concerns people’s right to determine what happens to *their own bodies* (rather than their prerogative to decide what is in the best interest of their child). On the other hand, such measures will arguably be called for only when there is an immediate threat of exceptional magnitude to society at large—COVID-19 has shown the societally devastating character of a pandemic.

15. We argue that in a pandemic, governments are justified in implementing policies that restrict individuals to certain places (pubs, restaurants, theatres, etc.) only if they are expected to be unable to transmit a particular pathogen, either because they have recently had a negative test result, or because they have gained immunity due to having the disease, or because they have been vaccinated.

16. In this case, proportionality is maintained by focusing only on contexts where the risks of transmission are high and by enabling individuals to access certain places with a negative test result, thereby avoiding vaccination. Of course, the exact features of a proportionate policy will depend on many contextual factors, including the vaccine’s effectiveness at producing sterilising immunity, the reliability of negative tests, and the necessity for individuals to have access to places or events with restricted access.

17. Any immunisation policy must ensure that people have reasons to trust that vaccines are effective and safe, and that the government takes their rights seriously. It is sometimes argued that adopting a mandatory policy will undermine trust in vaccination and thus be counterproductive. However, all Western countries that adopted mandatory policies in the past 10 years showed increased vaccine coverage in the years that followed.

18. The importance of trust underscores why a transparent and reasonable normative justification for vaccination policies is indispensable and why focusing on proportionality is so important.

19. However, we should not be concerned only with promoting trust among those who hold the majority view and are vaccine-hesitant. A democratic government must be considered trustworthy by all citizens, including those who justifiably expect the government to take its responsibility to protect the health of the population seriously and to implement effective measures when vaccine coverage falls below the previously specified minimum level.

20. The liberal paradigm of John Stuart Mill’s harm principle has often been criticised as too narrow to support effective public health policies. This critique, which calls for a greater role for values such as solidarity and care, is not unreasonable. However, by grounding mandatory vaccination in the harm principle, we offer the strongest possible justification for such a controversial policy: the restriction of liberty is ultimately justified by an appeal to the fundamental value of liberty itself. And this does not rule out embracing other values in a broader public health policy.

## References

[phag016-B1] Feinberg, J. (1984). The Moral Limits of the Criminal Law Volume 1: Harm to Others. New York: Oxford University Press.

[phag016-B2] Mill, J. S. (1859/2003). In Bromwich D. and Kateb G. (eds), On Liberty. New Haven: Yale University Press.

[phag016-B3] Pierik, R. and Verweij, M. (2024). Inducing Immunity? Justifying Immunization Policies in Times of Vaccine Hesitancy. Cambridge, MA: MITpress. 10.1093/phe/phag016 PMC13356940 42438464

